# FXYD6 is a new biomarker of cholangiocarcinoma

**DOI:** 10.3892/ol.2013.1727

**Published:** 2013-12-04

**Authors:** XIONGFEI CHEN, MINGZHU SUN, YAZHUO HU, HONGHONG ZHANG, ZHANBO WANG, NINGXIN ZHOU, XINYUN YAN

**Affiliations:** 1 Medical College of Soochow University, Industrial Park, Suzhou, Jiangsu 215123, P.R. China; 2 Department of Pathology, General Hospital of PLA Second Artillery, Beijing 100888, P.R. China; 3 Institute of Geriatrics, PLA General Hospital, Beijing 100853, P.R. China; 4 Department of Pathology, PLA General Hospital, Beijing 100853, P.R. China; 5 Institute of Hepatobiliary Gastrointestinal Disease, General Hospital of PLA Second Artillery, Beijing 100088, P.R. China; 6 Key Laboratory of Protein and Peptide Pharmaceuticals, CAS-University of Tokyo Joint Laboratory of Structural Virology and Immunology, Institute of Biophysics, Chinese Academy of Sciences, Beijing 100101, P.R. China

**Keywords:** cholangiocarcinoma, FXYD6, immunohistochemistry, differentiation

## Abstract

Members of the FXYD domain-containing ion transport regulator protein family, including FXYD3 and FXYD5, play an important role in the pathogenesis of numerous tumors. However, the correlation between the expression of FXYD6 and tumors remains poorly understood. In the current study, the expression of FXYD6 was examined immunohistochemically in 72 cholangiocarcinoma tissues and 30 distal normal bile duct tissues matched with the tumors. The results show that the positive expression rate of FXYD6 was significantly higher in cholangiocarcinoma than that in normal bile duct tissue (69 vs. 33.3%; P=0.002). Furthermore, the positive expression rate of FXYD6 in well- and moderately-differentiated cholangiocarcinoma was clearly higher than that in poorly-differentiated and mucinous cholangiocarcinoma (85.7 vs. 40%; P=0.000). However, there was no significant correlation between the expression of FXYD6 and gender (P=0.393), age (P=0.174), histological type (P=0.123), T stage (P=0.164), lymph node metastasis (P=0.343), perineural invasion (P=0.088) and tumor location (P=0.238). The results of this study indicate that FXYD6 may be a new biomarker for cholangiocarcinoma and may be associated with a favorable prognosis in this malignant disease.

## Introduction

Cholangiocarcinoma (CC), originating from bile duct epithelial cells, is a highly malignant tumor. It is resistant to conventional therapies and has a poor prognosis (1). CC may arise anywhere in the biliary tree, from the small peripheral hepatic ducts to the distal common bile duct. Based on its anatomical location, it is commonly divided into three categories: Intrahepatic (20–25%), perihilar (50–60%) and distal (20–25%) tumors (2). CC has a particularly poor prognosis, as the majority of patients are diagnosed at an advanced stage. Therefore, early and accurate diagnosis is essential. With advances in biotechnological techniques and a deepened understanding of the biological behavior of CC, specific markers have been studied, including TP53 gene mutation, cyclins and proliferation indices (3). However, to date, no characterized tumor markers have been validated for disease diagnosis. Hence, new biomarkers for validation and prognosis of CC are required.

FXYD domain-containing ion transport regulator 6 (FXYD6), also called phosphohippolin, is a new member of the FXYD protein family and a regulator of Na, K-ATPase (4). Previous studies have reported that specific members of the FXYD protein family, including FXYD3 and FXYD5, play important roles in the pathogenesis of a number of tumor types (5–8). Preliminary studies have shown that FXYD6 is highly expressed in the brain and plays an essential role in the excitability and development of neurons (9–10). However, the correlation between the expression of FXYD6 and tumors remains largely unknown. Olstad *et al*(11) originally reported that FXYD6 mRNA was upregulated in osteosarcoma target cell lines compared with normal osteoblasts, and our previous study also showed that FXYD6 mRNA was overexpressed in CC tissues compared with normal bile duct tissues (12), suggesting that FXYD6 may be involved in tumor initiation. To date, the expression of FXYD6 at the protein level and its clinical significance in human resected CC cases remains unclear. In the present study, the expression of FXYD6 was analyzed immunohistochemically in a series of 72 CC tissues along with 30 matched distal normal bile duct tissues. Furthermore, the clinicopathological significance of FXYD6 protein expression in CC is discussed.

## Materials and methods

### Clinical samples

The formalin-fixed paraffin-embedded tissue samples were obtained from 72 primary CC patients who underwent surgical resection in the General Hospital of PLA Second Artillery (Beijing, China) between 2007 and 2012. CC tissues were staged according to the TNM system defined by the World Health Organization (WHO) staging system. None of the patients received preoperative chemotherapy or radiotherapy. Thirty normal bile duct specimens distal to CC were matched with the primary tumor. The pathological slides, including normal and neoplastic specimens, were confirmed by pathology. Patient information, including gender, age, differentiation, histology type, T stage, lymph node metastasis, perineural invasion and tumor location (Table I) was obtained from surgical and/or pathological records at the hospital.

The mean age of the patients (44 male and 28 female) was 62.14±10.53 years (ranging between 30 and 83 years) and the median age was 62.5 years. Forty-two (58.3%) neoplastic specimens were well- or moderately-differentiated carcinomas, and 30 (41.7%) were poorly-differentiated carcinomas. According to the WHO histological classification system, the pathological subtypes present were 61 adenocarcinomas (84.7%), 9 papillocarcinomas (12.5%) and 2 mucinous carcinomas (2.8%). Intrahepatic CC occurred in 13 cases (18.1%), perihilar CC occurred in 41 cases (56.9%) and distal CC occurred in 18 cases (25%). Of the 59 extra-hepatic CC tissues there were 22 cases (37.3%) in T1–2 stage and 37 cases (62.7%) in T3 stage, according to WHO TNM system, and 24 cases (40.7%) with perineural invasion and 19 cases (32.2%) with lymph node metastasis. The project was approved by the ethics committee of General Hospital of PLA Second Artillery and written informed consent was obtained from all patients prior to enrollment.

### Immunohistochemistry

For each case, the specimens were fixed in 10% formalin, embedded in paraffin and serially sectioned at 4 μm. For immunohistochemistry, sections were incubated at 60°C, de-paraffinized and then rehydrated prior to being microwaved at 500 W for 15 min in 10 mM sodium citrate buffer (pH 6.0) for antigen retrieval. The slides were allowed to cool down naturally in the buffer at room temperature. Endogenous peroxidase activity was blocked by incubation with 0.3% H_2_O_2_ in methanol for 30 min, followed by washing with phosphate-buffered saline (PBS). Following blockage of non-specific reactions with 5% normal horse serum for 1 h, sections were incubated with monoclonal FXYD6 [generated by our lab (13)] overnight at 4°C in a moist chamber. After washing with PBS, sections were incubated with a biotinylated horse anti-mouse IgG antibody (ZB-2020; ZSGB-BIO, Beijing, China) for 40 min at 37°C, washed again with PBS, and incubated with horseradish peroxidase streptavidin (ZB-2404; ZSGB-BIO) for 40 min. The peroxidase reaction was developed in freshly prepared 3,3′-diaminobenzidine solutions (ZLI-9017; ZSGB-BIO) and observed under a microscope (BX53l Olympus, Tokyo, Japan). The sections were then rinsed in water, counterstained using hematoxylin, dehydrated in ethanol and mounted with xylene-based mounting medium. For negative controls, PBS was used instead of the primary antibody under the same conditions.

The expression of FXYD6 in CC and normal bile duct tissues was evaluated with whole slide scanning under low magnification (x40) and then confirmed under high magnification (×200 and ×400). An immunoreactivity scoring system was applied. The percentage of positively stained cells was used to determined the following scores: ≤5%, 0; 6–25%, 1; 26–50%, 2; 51–75%, 3; and >75%, 4. The score intensity of color staining was defined by the following parameters: Colorless, 0; whitish yellow, 1; yellow, 2; and brown, 3. The staining score was determined by multiplying the scores obtained from the percentage of stained cells and intensity of staining and was stratified as follows: − (0, absent); + ( 1–4, weak); ++ (5–8, moderate); and +++ (9–12, strong). An optimal cut-off value was identified: Cells with a final staining score of − or + were classified as FXYD6-negative, and cells with a final staining score of ++ or +++ were classified as FXYD6-positive. The slides were examined independently by two observers blinded to clinical and pathological data, and all discrepancies were resolved by joint review of the slides in question. To avoid artificial effects, tissues in areas with poor morphology and necrosis, and in the section margins, were not considered.

### Statistical analysis

The χ^2^ method and Fisher’s exact test were used to examine the correlation between clinicopathological characteristics of patients and the frequencies of FXYD6 expression in CC, using SPSS software (v13.0; SPSS Inc., Chicago, IL, USA). All P-values were two-tailed and P<0.05 was considered to indicate a statistically significant difference.

## Results

### FXYD6 is highly expressed in CC

**Figure 1:**
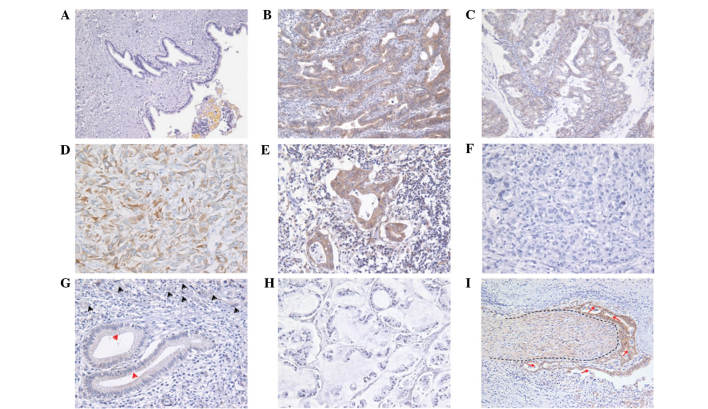
Representative immunohistochemical staining in normal bile mucosa and CC. (A) Negative signal of FXYD6 was detected in normal bile duct tissues. By contrast, moderate expression of FXYD6 was found in (B) well-differentiated papillocarcinoma and significant FXYD6 expression was found in (C) moderately-differentiated adenocarcinoma, (D) poorly-differentiated adenocarcinoma and (E) lymph node metastases. (F and G) Negative signal of FXYD6 was detected in bile duct (red arrow), poorly-differentiated CC (black arrow) and (H) mucinous carcinoma. (I) FXYD6 was widely distributed in the infiltrative nerve (the dotted area) and the CC cells (red arrow). Magnification, (A, B, C, H and I) ×200 and (D, E, F and G) ×400. CC, cholangiocarcinoma.

To clarify whether FXYD6 was highly expressed in CC, the mouse anti-human FXYD6 monoclonal antibody, generated from our laboratory, was used to immunohistochemically detect FXYD6 protein levels in 72 CC and 30 distal non-cancerous bile duct tissues. Negative immunostaining was observed in the majority of normal bile duct tissues: FXYD6 negative reactivity was observed in 20/30 (66.6%) normal slides (Table I). In CC tissues, the positive expression rate of FXYD6 was 41/61 (67.2%) for adenocarcinoma, 7/9 (77.8%) for papillocarcinoma and 0/2 (0%) for mucinous carcinoma. Overall, the positive expression rate of FXYD6 antigen in CC tissues was 48/72 (69%), which was significantly higher than that in normal tissues (33.3%). Positive staining was observed in the cytoplasm of the glandular cancer cells (Fig. 1). As shown in Fig. 1I, FXYD6 expression was also detected in the infiltrative nerve fibers.

### Expression of FXYD6 correlates with histological grade

**Figure 2:**
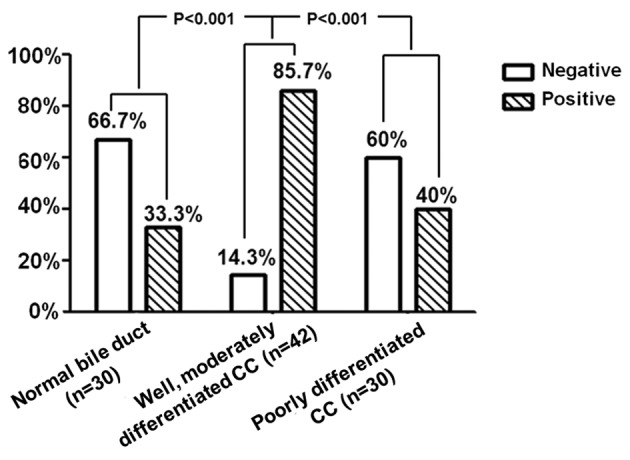
Expression of FXYD6 correlates with histological grade of CC. CC, cholangiocarcinoma.

The expression levels of FXYD6 in CC were observed to correlate with the degree of differentiation of the tumor. The expression of FXYD6 protein was examined in normal biliary mucosa and CC. Expression was identified in the cytoplasm of normal mucosa epithelial and cancer cells. The correlation between FXYD6 expression and various clinicopathological factors was also analyzed. As shown in Fig. 2, increased FXYD6 expression was found to significantly correlate with the degree of differentiation of CC: the positive expression rate of FXYD6 in well- and moderately-differentiated CC (36/42; 85.7%) was higher than that in poorly-differentiated CC (12/30; 40%). Intrahepatic CC specimens were limited to 13 cases in our study. Therefore, only T stage, lymph node metastasis and perineural invasion of extrahepatic CC were statistically analyzed. No significant correlation was found between FXYD6 expression and T stage (χ^2^=1.933; P=0.164), lymph node metastasis (χ^2^=0.899; P=0.343) and perineural invasion (χ^2^=2.910; P=0.088). There was also no significant correlation between the increased expression of FXYD6 and other clinicopathological factors, including gender (χ^2^=0.731; P=0.393), age (χ^2^=1.848; P=0.174), histological type (P=0.123) and tumor location (χ^2^=2.817; P=0.238).

## Discussion

CC is considered to be a highly fatal disease with a poor prognosis due to early invasion, widespread metastasis and a lack of effective therapy. However, no biomarkers have been identified for CC. Therefore, for the diagnosis and therapy of malignant tumors, it is imperative that effective prognostic biomarkers are found which underlie the progression.

The present study concentrated on proteins which function as ion channels and participate in intracellular or extracellular information transmission, particularly in the molecular regulation network, which leads to cellular oncogenesis. The FXYD protein family, known as a regulator of Na, K-ATPase, was named in recognition of invariant amino acids in its signature motif and includes seven members found in mammals (14). Two family members, FXYD3 (mammary tumor protein 8 kD) and FXYD5 (dyshaderin or resembles ion channel), are highly expressed in numerous malignant tumors and are associated with tumor cell invasion and migration, involvement of lymph nodes and prognosis (5–8). According to a preliminary study by Olstad *et al*(11) using directional tag PCR subtractive hybridization, FXYD6 mRNA was clearly upregulated in osteosarcoma target cell lines compared with normal osteoblasts. Furthermore, we have previously reported that FXYD6 mRNA was relatively increased in CC tissues compared with normal bile duct tissues (12), indicating that it may be involved in cellular carcinogenesis. However, there are no systematic studies on the molecule at the protein level. In the present study, 72 CC and 30 distal bile duct tissues were used to analyze the clinicopathological significance of FXYD6. The results show that the expression of FXYD6 protein is significantly associated with CC. The positive expression rate of FXYD6 protein in CC tissue was notably higher than that in distal bile duct tissue (69 vs. 33.3%; P=0.002), indicating that FXYD6 may be a new potential biomarker and therapeutic target for CC.

Furthermore, FXYD6 expression was also observed to be associated with tumor differentiation. As shown in Fig. 2, the positive rate of FXYD6 expression decreased with a higher histological grade. The positive expression rate of FXYD6 in poorly-differentiated carcinoma was significantly lower compared to well- and moderately-differentiated carcinoma (87.5 vs. 40%; P=0.000). There was no positive expression in the two mucinous carcinoma tissues (Fig. 1H). The histological grade of CC is an independent prognostic factor, with a poorly-differentiated tumor accompanied by a poorer prognosis for patients with CC (15). The prognosis of mucinous carcinoma is particularly poor (16), therefore, mucinous carcinoma and poorly-differentiated carcinoma were classified into one group in this study. However, there was no information on patient follow-up in the present study and statistical analysis of the correlation between FXYD6 expression and survival was not performed. Due to the poor prognosis in poorly-differentiated CC compared with well- and moderately-differentiated CC, we hypothesize that increased expression of FXYD6 protein may be associated with a favorable prognosis in CC. Usually, it is difficult to distinguish hyperplastic bile ducts from well-differentiated CC. Due to the obstruction of the biliary tract and cholestasis, the bile duct above the site of obstruction frequently undergoes inflammatory proliferation, and it is difficult to pathologically distinguish heteromorphic CC from inflammatory proliferation. In the present study, the positive expression rate of FXYD6 in well- and moderately-differentiated CCs was statistically higher than that in distal bile duct tissue (87.5 vs. 33.3%; P=0.000). It may be useful for pathologists to differentially diagnose inflammatory proliferation and CC cases with higher levels of differentiation through the detection of FXYD6 expression.

In previous studies, FXYD6, a new member of the FXYD protein family, has been shown to be present in neuronal cells, but not in glial cells (9). This membrane protein is also important in the excitability and development of neurons (9–10). In the present study, FXYD6 protein expression was widely distributed in nerve fibers (Fig. 1H), consistent with the observations of Kadowaki *et al*(9). With regard to the association between the FXYD6 gene and schizophrenia, no consistent conclusion has been reached (17–19). Perineural invasion is a common pathway for CC metastasis and correlates with postoperative recurrence and poor prognosis (20). In the current study, FXYD6 was not only expressed in nerve fibers but also in the majority of CC cells. We hypothesize that the protein may be associated with perineural invasion, however, the result was not significant (χ^2^=2.910; P=0.088). By contrast, there was a positive correlation between the expression of FXYD6 in CC and perineural invasion. One explanation for this result is that specific perihilar CC specimens were locally excisional, and tissues with infiltration of the nerves may not have been selected. Therefore, given these material constraints, further studies are required in order to determine the correlation between the expression of FXYD6 and perineural invasion.

FXYD6 is a new regulator of Na, K-ATPase. Delprat *et al*(4) have reported that FXYD6 modulates the Na, K-ATPase transport properties and plays an essential role in endolymph production and/or endolymph endocochlear potential generation in the inner ear. Shindo *et al*(21) have revealed that FXYD6 is frequently co-expressed with the Na, K-ATPase β1 subunit in type II taste cells, which indicates that FXYD6 participates in olfactory signal transduction in type II taste cells through the modulation of the β1 subunit. The α1 and β1 subunits of Na, K-ATPase are markedly associated with carcinoma and have become the treatment target for specific tumors (22–25). Based on the aforementioned data, we hypothesize that FXYD6, as a novel modulator of Na, K-ATPase, is involved in the malignant transformation of biliary tract epithelia through the regulation of the α1 or β1 subunit of Na, K-ATPase. The mechanism of FXYD6 overexpression in CC is not understood and requires further exploration. The significant differences in FXYD6 expression in normal bile duct and CC tissues indicate that this protein may play an important role in carcinogenesis, for example tumor initiation. In addition, since FXYD6 was observed to be more frequently expressed in CC with higher differentiation, it may participate in CC progression. In conclusion, the results of this study indicate that FXYD6 may be a new biomarker for CC, particularly in tumors with a low histological grade.

## Figures and Tables

**Table I:** Correlation between FXYD6 protein expression and clinicopathological variables in patients with CC.

		FXYD6 expression			
				
Variables	n	Negative (%)	Positive (%)	χ^2^	P-value
Tissue type				9.592	0.002
Normal	30	20 (66.7)	10 (33.3)		
Carcinoma	72	24 (31)	48 (69)		
Gender				0.731	0.393
Male	44	13 (29.5)	31 (70.5)		
Female	28	11 (39.3)	17 (60.7)		
Age, years				1.848	0.174
≤60	29	7 (24.1)	22 (75.9)		
>60	43	17 (39.5)	26 (60.5)		
Differentiation^a^				16.457	0.000
Well, mod	42	6 (14.3)	36 (85.7)		
Poor	30	18 (60)	12 (40)		
Histological type					0.123^c^
Adencarcinoma	61	20 (32.8)	41 (67.2)		
Papillocarcinoma	9	2 (22.2)	7 (77.8)		
Mucinous carcinoma	2	2 (100)	0		
T stage^b^				1.933	0.164
T1–2	22	4 (18.2)	18 (81.8)		
T3	37	13 (35.1)	24 (64.9)		
Lymph node metastasis^b^				0.899	0.343
Negative	30	20 (50)	20 (50)		
Positive	19	7 (36.8)	12 (63.2)		
Perineural invasion^b^				2.910	0.088
Negative	35	13 (37.1)	22 (62.9)		
Positive	24	4 (16.7)	20 (83.3)		
Location				2.817	0.238
Intrahepatic	13	7 (53.8)	6 (46.2)		
Perihilar	41	12 (29.3)	29 (70.7)		
Distital	18	5 (27.8)	13 (72.2)		

a Well, well-differentiated adenocarcinoma; mod, moderately-differentiated adenocarcinoma; poor, poorly-differentiated adenocarcinoma. Mucinous carcinoma and poorly differentiated adenocarcinoma were classified into a group due to the analogously bad prognosis.

b Refers to extra-hepatic CC.

c Fisher’s exact test.

CC, cholangiocarcinoma.
